# Fast and efficient Rmap assembly using the Bi-labelled de Bruijn graph

**DOI:** 10.1186/s13015-021-00182-9

**Published:** 2021-05-25

**Authors:** Kingshuk Mukherjee, Massimiliano Rossi, Leena Salmela, Christina Boucher

**Affiliations:** 1 Department of Computer and Information Science and Engineering, Herbert Wertheim College of Engineering, University of Florida, Gainesville, USA; 2 Department of Computer Science, Helsinki Institute for Information Technology, HIIT, University of Helsinki, Helsinki, Finland

**Keywords:** Optical mapping, Single molecule maps, de Bruijn graph, Overlap-layout-consensus, Genome assembly, Mis-assemblies

## Abstract

Genome wide optical maps are high resolution restriction maps that give a unique numeric representation to a genome. They are produced by assembling hundreds of thousands of single molecule optical maps, which are called Rmaps. Unfortunately, there are very few choices for assembling Rmap data. There exists only one publicly-available non-proprietary method for assembly and one proprietary software that is available via an executable. Furthermore, the publicly-available method, by Valouev et al. (Proc Natl Acad Sci USA 103(43):15770–15775, 2006), follows the overlap-layout-consensus (OLC) paradigm, and therefore, is unable to scale for relatively large genomes. The algorithm behind the proprietary method, Bionano Genomics’ Solve, is largely unknown. In this paper, we extend the definition of bi-labels in the paired de Bruijn graph to the context of optical mapping data, and present the first de Bruijn graph based method for Rmap assembly. We implement our approach, which we refer to as rmapper, and compare its performance against the assembler of Valouev et al. (Proc Natl Acad Sci USA 103(43):15770–15775, 2006) and Solve by Bionano Genomics on data from three genomes: *E. coli*, human, and climbing perch fish (*Anabas Testudineus*). Our method was able to successfully run on all three genomes. The method of Valouev et al. (Proc Natl Acad Sci USA 103(43):15770–15775, 2006) only successfully ran on *E. coli*. Moreover, on the human genome rmapper was at least 130 times faster than Bionano Solve, used five times less memory and produced the highest genome fraction with zero mis-assemblies. Our software, rmapper is written in C++ and is publicly available under GNU General Public License at https://github.com/kingufl/Rmapper.

**Supplementary Information:**

The online version contains supplementary material available at 10.1186/s13015-021-00182-9.

## Introduction

In 1993 Schwartz et al. developed *optical mapping* [1], a system for creating an ordered, genome wide high resolution restriction map of a given organism’s genome. Since this initial development, genome wide optical maps have found numerous applications including discovering structural variations [2, 3], scaffolding and validating contigs for several large sequencing projects [4, 5], and detecting mis-assembled regions in draft genomes [6–8]. Thus, optical mapping has assisted in the assembly of a variety of species – including various prokaryotic species [9–11], rice [12], maize [13], mouse [14], goat [15], parrot [4], and *amborella trichopoda* [5]. Bionano Genomics has enabled the automated generation of the data, enabling the data to become more wide-spread. For example, Bionano data was generated for 133 species sequenced for the Vertebrate Genomes Project.

Similar to sequencing, the protocol for producing optical mapping data, begins with many fragmented copies of the genome of interest. This redundancy allows overlap between the raw data and assembly into longer contiguous regions corresponding to the genome. With a selected enzyme, the genomic DNA fragments are nicked at each restriction site recognized by the enzyme. These cleaved fragments are then photographed and analyzed in order to determine the length (in kbp) of the regions between nick sites. The result of this process are optical maps for all the fragments, which are referred to as *Rmaps*. For example, given a genome fragment TTTTAACTGGGGGGGAACTTTTTTTTAACTTTTT and an enzyme that recognizes the site AACT and cleaves in the middle, the resulting Rmap would be [6, 11, 11, 6]. Rmaps by themselves are not traditionally used for analysis—although, they can be [2, 3, 16]—and instead have to be assembled into longer contiguous optical maps corresponding to the genome. Hence, assembly of Rmaps refers to the problem of generating a consensus genome wide optical map from overlapping Rmaps.

Although optical mapping has been around for several decades, the problem of efficiently assembling the data largely remains open as there has been little work in this area—which is largely due to the challenges posed by the data itself. We should note that several related problems, such as alignment of optical mapping data [16–22], have been more thoroughly explored. Rmap data has a number of errors that make it difficult to assemble—namely, there exists added and deleted cut sites and sizing error, resulting in extra fragments, merges in neighboring fragments and under or over-estimates of the length of a fragment. In the running example, the error free Rmap of [6, 11, 11, 6] could occur as [6, 22, 6] with error. Nonetheless, there exists two Rmap assembly methods: Gentig by Anantharaman et al. [23] and the assembler of Valouev et al. [24]. Developed in 1998, Gentig is the first Rmap assembly algorithm. It is based on a Bayesian model that seeks to maximize the *a posteriori* estimate of the consensus optical map produced by the assembly of Rmaps. It first computes the overlap between all pairs of Rmaps using dynamic programming, and then builds contigs by greedily merging the Rmaps based on alignment score. This process of merging contigs continues until all alignments above a certain score are merged. Valouev et al. [24] implemented an overlap-layout-consensus (OLC) assembly algorithm using their alignment algorithm [25], which also starts by calculating alignment between all pairs of Rmaps, and identifying all alignments that have score above a specified threshold. A graph is built, where Rmaps are represented as nodes, and the non-filtered alignments are represented as edges. The graph is refined by eliminating paths in the graph that are weakly supported. In other words, if two connected regions in the graph are joined by only a single path—or with multiple paths, but having one or more common intermediate nodes—then the graph is disconnected at these nodes. Further, an edge is removed if it is inconsistent with a higher scoring edge. Contigs are then generated by traversing this graph in a depth first manner. Bionano Genomics Inc. provides a proprietary assembly method, called Bionano Solve, however the source code is not publicly available and the algorithmic details are unknown due to the proprietary nature of the software.

The alternative to an OLC approach for assembly is a de Bruijn graph approach that relies on building and traversing a de Bruijn graph constructed on the sequence data. For simplicity, we give a constructive definition of the de Bruijn graph in the context of genome assembly. Given a set of sequences $R = \{r_1,\ldots , r_n \}$ and an integer *k*, the de Bruijn graph is constructed by creating a directed edge for each unique *k* length substring (*k*-mer) with the nodes labeled as the $k - 1$ length prefix and $k - 1$ length suffix of the *k*-mer, and then all nodes that have the same label are merged. The important aspect of the de Bruijn graph assembly approach is that it avoids having to find alignments between any pair of sequences, leading to an ${\mathcal {O}}(n)$ run-time. Since its introduction by Idury et al. [26] and Pevzner et al. [27], this approach has become the most common paradigm for assembling short read sequencing data because it led to huge gains in performance over OLC approaches. Hence, applying a de Bruijn graph approach to Rmap assembly would likely lead to similar improvements by removing the burden of finding all pairwise alignments between Rmaps. This assembly works on the premise that a *k*-mer will occur exactly without error frequently in the data. Hence, the biggest challenge we face is constructing a de Bruijn graph with added and deleted cut-sites and sizing error. Even without the occurrence of added and deleted cut-sites, *k*-mers created from Rmap data are unlikely to be exact replicas due to sizing error. For example, [6, 11, 11, 6] and [5, 10, 11, 7] should likely be recognized as instances of the same *k*-mers in Rmap data. Thus, to overcome this challenge the de Bruijn graph has to be redefined to account for the inexactness of the data.

In this paper, we formulate and describe a de Bruijn graph approach for *de novo* Rmap assembly, which heavily relies on redefining the de Bruijn graph to make it suitable for Rmap data. We accomplish this by extending the definition of a bi-label in the context of the paired de Bruijn graph that was introduced by Medvedev et al. [28]. We refer to our modified de Bruijn graph as *bi-labelled de Bruijn graph*. Next, we demonstrate how to efficiently build and store the de Bruijn graph using a two tier orthogonal-range search data structure. We implement this approach, leading to a novel Rmap assembler that we call ${\textsc {rmapper}}$. We compare the performance of our method with the assembler of Valouev et al., and Bionano Solve on three genomes of varying size: *E. coli*, human, climbing perch (a fish species from the Vertebrate Genomes Project). Our comparison demonstrates that ${\textsc {rmapper}}$ was more than 130 times faster and used less than five times less memory than Solve, and was more than 2,000 times faster than Valouev et al. Also, ${\textsc {rmapper}}$ successfully assembled the 3.1 million Rmaps of the climbing perch genome into contigs that covered over 95% of the draft genome with zero mis-assemblies.

## Background and definitions

### Rmap data and genome wide optical maps

From a computer science perspective, we can view an Rmap $R=[r_1, r_2, \dots , r_{|R|}]$ as an ordered list of integers. Each number represents the length of the respective fragment. The *size* of an Rmap *R* denotes the number of fragments in *R*, which we denote as |*R*|. For example, say we have an enzyme that cleaves the DNA at the middle position of AACT and a genomic sequence TTTTAACTGGGGGGGAACTTTTTTTTAACTTTTT, then the Rmap will be $R = [6, 11, 11, 6]$ corresponding to the cleaved sequences [TTTTAA, CTGGGGGGGAA, CTTTTTTTTAA, CTTTTT].

### Error profile of Rmap data

There are three types of errors that can occur in optical mapping: (1) missing cut sites which are caused by an enzyme not cleaving at a specific site, (2) additional cut sites which can occur due to random DNA breakage and (3) inaccuracy in the fragment size due to the inability of the system to accurately estimate the fragment size. Continuing again with the example above, an example of an additional cut site would be when the second fragment of *R* is split into two, e.g., $R' = [6, 5, 6, 11, 6]$, and an example of a missing cut site would be when the last two fragments of *R* are joined into a single fragment, e.g., $R' = [6, 11, 17]$. Lastly, an example of a sizing error would be if the size of the first fragment is estimated to be 7 rather than 6.

Several different probabilistic models have been proposed for describing the sizing error, and the frequency of added and missed cut-sites, including the models of Valouev et al. [25], Li et al. [29], and Chen et al. [30]. We briefly describe these models here but refer to the original papers for a full description. Both Valouev et al. and Chen et al. describe the observed fragment lengths as normal distribution with the mean being equal to the true length of the fragment and the standard deviation being a function of the true length, i.e. longer fragments exhibit larger standard deviation. In the model by Li et al. the sizing error uses a Laplace distribution as follows: if the observed and actual size of a fragment are $o_i$ and $r_i$, respectively, then the sizing error, $o_i \sim r_i \times Laplace (\mu ,\beta )$ where $\mu$ and $\beta$ are parameters of the Laplace distribution and are functions of $r_i$. All studies model the probability of having a missed cut-site as a Bernoulli trial. Valouev et al. and Chen et al. predict a fixed probability for digestion of a cut-site while Li *et al.* model the probability of digestion as a function of lengths of the fragments flanking the cut-site. The likelihood of a missed cut-site decreases with the length of the fragment. All three models postulate additional or false cut-sites result from random breaks of the DNA molecule and hence model the number of false cuts per unit length of DNA as a Poisson distribution. Li *et al.* observed that false cuts occurred less frequently at the two ends of an Rmap.

### Rmap segments and k-mers

We define a *segment* $s_{p,q}$ of an Rmap starting at position *p* and ending at position *q*, as the $q-p+1$ consecutive fragments starting from $r_p$, i.e., $[r_{p}, r_{p + 1},.., r_{q}]$. We define the *length* of a segment as the summation of all of its constituent fragments, i.e., $r_p + \dots +r_q$. We denote the length of a segment $s_{p,q}$ as $\ell (s_{p,q})$. We note that the length of the Rmap *R* should not be confused with the number of fragments, which we denote as its size |*R*|.

In this paper, we extend the definition of a *k*-mer to the context of Rmap data as follows. Given an integer *k*, we define a *k*-mer as a segment of exactly *k* fragments, i.e., a sequence of *k* successive fragments of an Rmap. Following the example from above, the following two 3-mers exist in $R = [6, 11, 11, 6]$: [6, 11, 11] and [11, 11, 6].

### Prefixes and suffixes of Rmaps

Given an Rmap $R=[r_1, r_2, \dots , r_{|R|}]$, we define the *x*-size *prefix* of *R* as $R=[r_1, r_2, \dots , r_x]$, where *x* is at most $|R| - 1$. Conversely, we define the *x*-size *suffix* of *R* as $R=[r_{|R|-x+1}, \dots , r_{|R|}]$, where *x* is at most $|R| - 1$.

## The Bi-labelled de Bruijn graph

In this section, we modify the traditional definition of the de Bruijn graph for Rmap data by first redefining the concept of a bi-label for Rmap data. The term bi-label was first introduced by Medvedev et al. [28] in the context of short read assembly to incorporate mate-pair data into assembly of paired-end reads. There the term bi-label refers to two *k*-mers separated by a specified genomic distance. The redefinition of the de Bruijn graph with this extra information was shown to de-tangle the resulting graph, making traversal more efficient and accurate. Here, we demonstrate that an equivalent paradigm can be effective for Rmap assembly.

### Bi-labels

Given integers *k* and *D*, and Rmap *R*, we define a *bi-label* from an Rmap *R*, as a segment of *R* containing a pair of *k*-mers separated by the shortest segment that has a length of at least *D*. The following is a formal definition.

#### **Definition 1**

Given an Rmap $R = [r_1, r_2,..., r_i, r_{i + 1},.., r_{|R|}]$, integers *k* and *D*, and a position *i*, we define the bi-label at position *i* to be $[s_k^1, r_{p}, \dots , r_{q}, s_k^2]$, where $p=i + k$ and *q* is an index such that $\ell (s_{p,q-1}) < D \le \ell (s_{p,q})$

and $s_k^1$ and $s_k^2$ are the *k*-mers starting at positions *i* and $q+1$, respectively.

Next, we refer to segment $s_{p,q}$ between $s_k^1$ and $s_k^2$ as the *skip segment*, and note that, unlike $s_k^1$ and $s_k^2$ which both have *k* fragments, this segment is only bounded by its length and can have any number of fragments. Thus, this accounts for added and deleted cut-sites since these errors do not impact the length of a segment. Figure 2 demonstrates how the skip-segment tolerates a deleted cut-site. For example, given $k=3$, $D = 25$, and $R=[7,18,13,3,15,12,4,3,6,5,13,2]$, the bi-labels of *R* are $\Big ([7,18,13] \Big | [3,15,12]\Big |[4,3,6]\Big )$, $\Big ([18,13,3]\Big |[15,12]\Big |[4,3,6]\Big )$ and $\Big ([13,3,15]\Big |[12,4,3,6]$ $\Big |[5,13,2]\Big )$. We are now going to define the prefix and suffix bi-labels.

#### **Definition 2**

Given integers *D* and *k* and bi-label *b* with *k*-mers $b^1 =[b^1_1,..b^1_k]$ and $b^2 = [b^2_1,..,b^2_k]$ and skip segment $b^s$, we define the *prefix bi-label* of *b* as the bi-label with $(k-1)$-mers and skip-segment length at least *D*, where the first $(k-1)$-mer is the $(k-1)$-size prefix of $b^1$ i.e. $[b^1_1,..b^1_{k -1}]$.

Note that the second $(k-1)$-mer of the prefix bi-label is not necessarily the $(k-1)$-size prefix of $b^2$. We also require an equivalent definition for the suffix of a bi-label.

#### **Definition 3**

Given integers *D* and *k* and bi-label *b* with *k*-mers $b^1 =[b^1_1,..b^1_k]$ and $b^2 = [b^2_1,..,b^2_k]$ and skip segment $b^s$, we define the *suffix bi-label* of *b* as the bi-label with $(k-1)$-mers and skip-segment length at least *D*, where the first $(k-1)$-mer is the $(k-1)$-size suffix of $b^1$ i.e. $[b^1_2,..b^1_{k}]$.

**Fig. 1:**
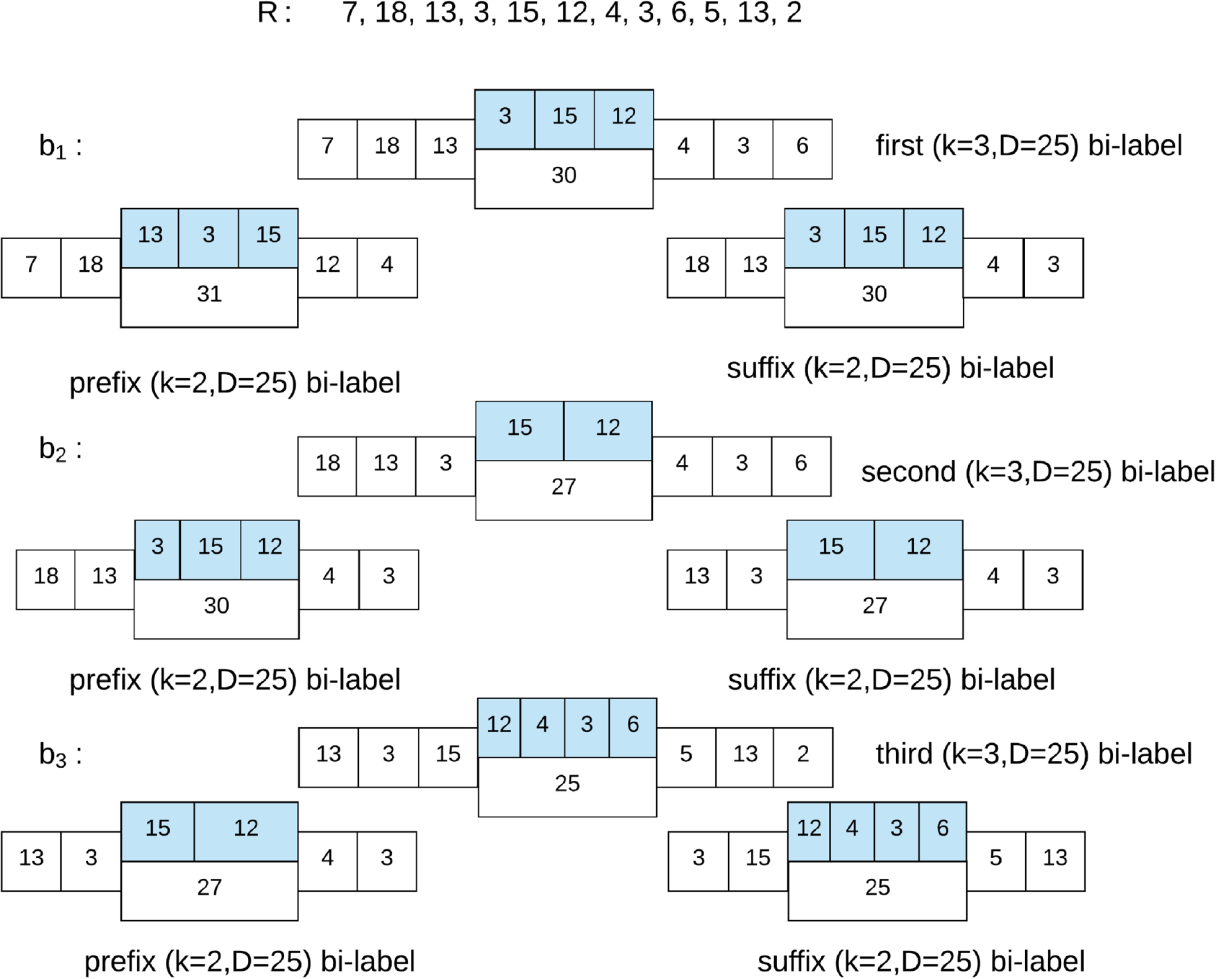
All bi-labels for $k=3$ and $D=25$ of an Rmap *R*. On each bi-label the fragments from the *k*-mers and the length of the skip segment are shown in white while the fragments of the skip segment are shown in blue. For each bi-label we show the prefix and suffix bi-labels built with $k=2$ and $D=25$

Figure 1 illustrates this concept of prefix and suffix bi-labels. Note that for two successive bi-labels from an Rmap, the prefix bi-label of the latter is the same as the suffix bi-label of the former as shown in Fig. 1. This is a vital property that allows the de Bruijn graph constructed over bi-labels to be connected.

**Fig. 2:**
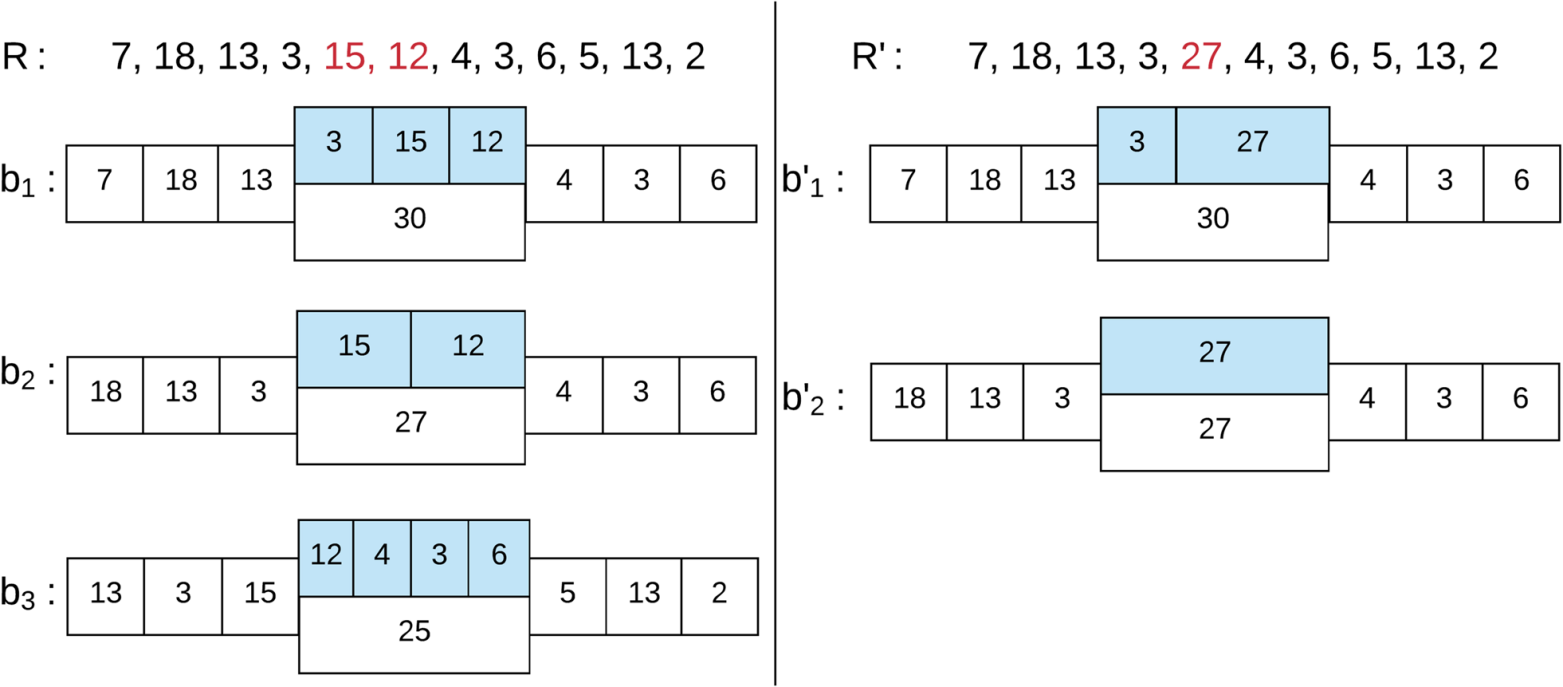
Skip segment overcomes missed cut-site. All bi-labels for $k=3$ and $D=25$ of two Rmaps *R* and $R'$, $\{b_1,b_2,b_3\}$ and $\{b'_1,b'_2\}$ respectively. Both Rmaps cover the same genomic location but $R'$ has a missed cut-site in position 5 (shown in red). On each bi-label the fragments from the *k*-mers and the length of the skip segment are shown in white while the fragments of the skip segment are shown in blue. Despite the missed cut-site on $R'$ bi-labels $b_1$ and $b_2$ are merged to $b'_1$ and $b'_2$ respectively according to our merge function

### Bi-label proximity

One of the challenges with Rmap data is the fact that the fragments correspond to genomic distances and due to experimental error, the measured estimates for the same genomic fragment are different across different Rmaps representing the same genomic location. For example, $R = [5, 6, 7, 11, 5]$ and $R' = [6, 5, 6, 11, 6]$ likely correspond to the same *k*-mer but the numerical nature makes it such that they are not exactly equal. Thus, we need to define a criteria such that two bi-labels drawn from different Rmaps but corresponding to the same genomic locations can be identified and merged for the construction of the de Bruijn graph. Thus, to make the definition of a bi-label robust to sizing errors, we define conditions on both the difference of the individuals fragments of two bi-labels and the difference in the total lengths. Hence, we have the following definitions.

#### **Definition 4**

Given integers $t_f$, *k* and *D*, and two bi-labels *a* and *b*, we let the *k*-mers of *a* and *b* be $a^1 = [a^1_1,.., a^1_k ]$ and $a^2 = [a^2_1,.., a^2_k ]$ and $b^1 = [b^1_1,.., b^1_k ]$ and $b^2 = [b^2_1,.., b^2_k ]$, respectively. We define *a* and *b* to be *fragment proximal* if and only if $|a^1_i - b^1_i| \le t_f$ and $|a^2_i - b^2_i| \le t_f$ for all $i = 1,..,k$.

Here $t_f$ is an error-tolerance parameter that handles sizing errors on the fragments of the bi-label.

#### **Definition 5**

Given integers $t_{\ell }$, *k* and *D*, and two bi-labels *a* and *b*, we let the *k*-mers of *a* and *b* be $a^1$ and $a^2$ and $b^1$ and $b^2$, respectively, and the skip segment of *a* and *b* be $a^s$ and $b^s$, respectively. We define *a* and *b* to be *length proximal* if and only if $|\ell (a^1) - \ell (b^1)| \le t_{\ell }$, $|\ell (a^2) - \ell (b^2)| \le t_{\ell }$ and $|\ell (a^s) -\ell (b^s)| \le t_{\ell }$.

Here $t_{\ell }$ is another error-tolerance parameter that handles sizing errors on the segment lengths of the bi-label. These two definitions lead to our final definition that defines whether two bi-labels should be defined as equivalent in the de Bruijn graph.

#### **Definition 6**

Given integers *k* and *D* and two bi-labels *a* and *b*, we define them to be *proximal* if and only if they are fragment proximal and length proximal.

This leads to our final definition, which is the set of bi-labels in which the bi-labelled de Bruijn graph is defined on.

#### **Definition 7**

Given a set of Rmaps $\{R_1,..,R_n\}$ and integers *k* and *D*, let *B* be the set of bi-labels from *R*. We define the *proximal reduced* set of bi-labels as the set $B'$, where for each *b* in *B* there is a bi-label in $B'$ that it is proximal to.

### Definition of the bi-labelled de Bruijn graph

Given the above definitions, we are now ready to define the bi-labelled de Bruijn graph built on a set of proximal bi-labels extracted from Rmaps.

#### **Definition 8**

Given integers *k* and *D* and set of Rmaps $\{R_1,.., R_n \}$, let *B* be the proximal reduced set of bi-labels extracted from *R*. We create a directed edge *e* for each bi-label *b* in *B* and label the incoming and outgoing nodes of *e* as the prefix bi-label of *b* and suffix bi-label of *b*, respectively. After all edges are formed, the graph undergoes a gluing operation. A pair of node bi-labels are glued into a single node if and only if they are proximal. We define the final graph obtained after gluing of nodes as the bi-labelled de Bruijn graph.

## Methods

In this section, we describe our method for building and traversing the bi-labelled de Bruijn graph from an Rmap dataset. Our method, which we refer to as ${\textsc {rmapper}}$, can be summarized into the following steps: extract and store bi-labels, find proximal bi-labels, build the bi-labelled de Bruijn graph, resolve tips and bubbles, and traverse the graph to build the contigs. We now describe each of these steps in detail.

### Extract and store all Bi-labels

We first error correct the Rmap data using cOMet [31] and then extract and store all bi-labels from the error corrected Rmaps. We recall from Definition 6 that two bi-labels are proximal if they are both fragment proximal as well as length proximal for error-tolerance parameters $t_f$ and $t_{\ell }$. Therefore, we must store all the bi-labels in a manner that allows finding all proximal bi-labels of a given bi-label efficiently. To accomplish this, we store all the bi-labels in a disjoint set of k-d trees [32] such that each pair of bi-labels in the same k-d tree is length proximal. For each bi-label, the 2*k* fragments of the *k*-mers of it are stored in the corresponding k-d tree, which will allow for efficiently finding all fragment proximal bi-labels of a given bi-label. Hence, the dimension of each k-d tree is 2*k*.

More formally, we identify each k-d tree ${\mathcal {K}}_{a_1,a_2,a_3}$ by three positive integers $a_1$, $a_2$, and $a_3$, and insert a given bi-label *b* into ${\mathcal {K}}_{a_1,a_2,a_3}$ if the length of its two *k*-mers $\ell (b^1)$ and $\ell (b^2)$ are within the range $[a_1 \times t_{\ell } ,\ldots , (a_1 + 1)\times t_{\ell } -1]$ and $[a_2 \times t_{\ell },\ldots , (a_2 + 1)\times t_{\ell } -1]$ respectively and the length of the skip segment $\ell (b^s)$ is also within the range $[a_3\times t_{\ell },\ldots , (a_3 + 1) \times t_{\ell }-1]$. If such a tree does not exist then we create a new one with ${\mathcal {K}}_{a_1,a_2,a_3}$, where $a_1=\lfloor {\ell (b^1)/t_{\ell }}\rfloor$, $a_2=\lfloor {\ell (b^2)/t_{\ell }}\rfloor$ and $a_3=\lfloor {\ell (b^s)/t_{\ell }}\rfloor$.

Next, for each bi-label in our set of k-d trees, we find and store pointers to all proximal bi-labels by performing an orthogonal range query. Given a bi-label *b* in ${\mathcal {K}}_{a_1,a_2,a_3}$, we let the *k*-mers of the bi-label *b* be $b^1 = [b^1_1,.., b^1_k ]$ and $b^2 = [b^2_1,.., b^2_k ]$. We perform a range query with $([{b_1^1}\pm t_f],\ldots , [{b_k^1}\pm t_f], [{b_1^2}\pm t_f],\ldots , [{b_k^2}\pm t_f])$ in the disjoint set of k-d trees to find all bi-labels whose first *k*-mer is equal to $[{b_1^1}\pm t_f],\ldots , [{b_k^1}\pm t_f]$ and whose second *k*-mer is equal to $[{b_1^2}\pm t_f],\ldots , [{b_k^2}\pm t_f]$. We add a pointer from *b* to each of these bi-labels. We repeat this for each bi-label. In particular, we perform the range query in all k-d trees where the proximal bi-labels can be found, i.e., all k-d trees ${\mathcal {K}}_{a'_1,a'_2,a_3}$ where for $m=min(k t_f, t_{\ell })$ we have, $\lfloor {(\ell (b^1) - m)/t_{\ell }}\rfloor \le a'_1 \le \lfloor {(\ell (b^1) + m)/t_{\ell }}\rfloor$ and $\lfloor {(\ell (b^2) -m)/t_{\ell }}\rfloor \le a'_2 \le \lfloor {(\ell (b^2) + m)/t_{\ell }}\rfloor$.

We note that k-d trees support multi-dimensional orthogonal range-search queries in ${\mathcal {O}}(n^{(2k-1)/2k}+occ)$ time and ${\mathcal {O}}(n)$ space where *n* is the number of bi-labels in the tree, *k* is the *k*-mer value, and *occ* is the number of bi-labels that satisfy the constraints of the range-search query.

### Graph construction

We first filter all low frequency bi-labels, i.e., bi-labels that have a low number of proximal bi-labels. As illustrated in Fig. 4, bi-labels that have low frequency typically arise from Rmap data that is highly erroneous. After filtering low frequency bi-labels, we build the bi-labelled de Bruijn graph by first building a proximal reduced set from the unfiltered bi-labels, then building all directed edges with labelled nodes from the reduced set, and finally merging nodes that have the same label. Using an efficient heuristic, we first greedily find the proximal reduced set of bi-labels by sorting the unfiltered bi-labels in descending order based on the number of proximal bi-labels found for them. From this sorted list of bi-labels *B*, we iteratively insert bi-labels into the reduced set $B'$ unless the bi-label is proximal to a bi-label already in $B'$.

Next, we build a bi-labelled de Bruijn graph by creating a directed edge for each bi-label $b'$ in $B'$ and labeling the incoming and outgoing nodes as the prefix bi-label and suffix bi-label of $b'$. We store all the nodes and edges in a modified adjacency list format that contains three arrays: one array stores all node bi-labels, one array containing a list of pointers of the incoming nodes for each node, and lastly, one array containing a list of pointers of the outgoing nodes for each node. Thus, to insert $b'$ into the graph, we first determine if the prefix and suffix bi-labels are contained in the node array and insert them if they are not contained in the list, and then insert an entry into the incoming and outgoing arrays with lists containing pointers to the prefix and suffix bi-labels. This graph representation will allow for the adjacency lists of two nodes to be efficiently merged if the bi-labels they represent are found to be proximal.

**Fig. 3:**
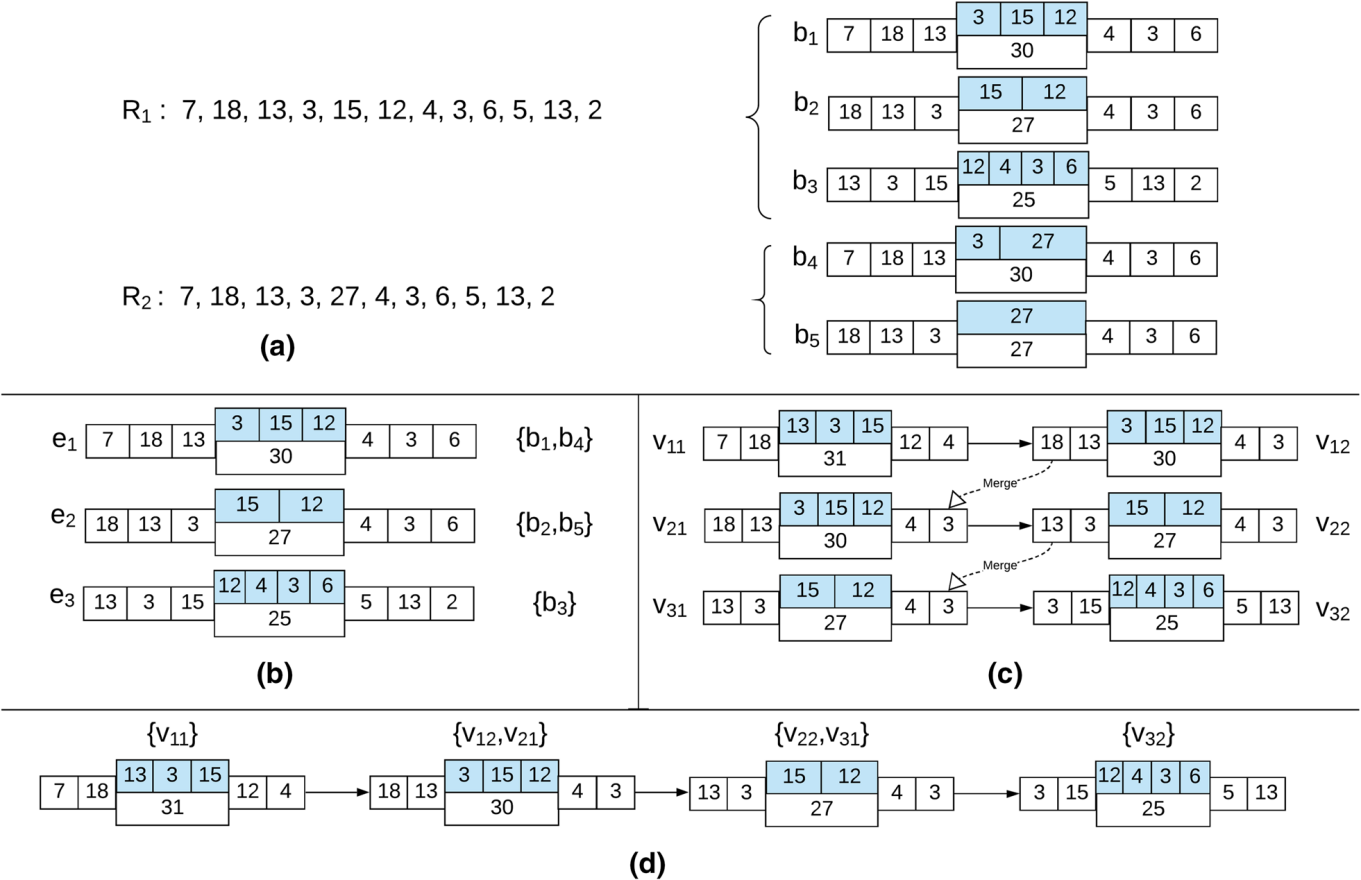
The construction of the bi-labelled de Bruijn Graph. **a** Two Rmaps $R_1$ and $R_2$ and the bi-labels extracted from them—$\{b_1,b_2,b_3\}$ from $R_1$ and $\{b_3,b_4\}$ from $R_2$ for $k=3$ and $D=25$. **b** Edges $\{e_1,e_2,e_3\}$ depict the proximal reduced set of bi-labels. Bi-labels $\{b_1,b_4\}$ are represented by $e_1$, bi-labels $\{b_2,b_5\}$ are represented by $e_2$ and bi-label $\{b_3\}$ forms $e_3$. We note that in this example no bi-labels are filtered for finding the proximal reduced set. **c** Nodes introduced into the graph. Each edge breaks into two nodes—one denoted by the prefix bi-label and the other by suffix bi-label of the edge. A directed edge is drawn from the former to the latter. **d** The final graph is formed by merging nodes $v_{12}$ with $v_{21}$ and merging $v_{22}$ with $v_{32}$

Lastly, we merge all nodes in the graph whose bi-labels are proximal to obtain the final bi-labelled de Bruijn graph. For merging the nodes, we again use a set of disjoint k-d trees as we did before for finding proximal bi-labels for the edge bi-labels. Hence, we extract all the node bi-labels and construct a set of k-d trees as before. Then for each node *v* in the node array, we query the corresponding k-d trees to find all nodes that are proximal to it using the same error tolerance parameters $t_f$ and $t_{\ell }$. Any node *u* that is found to be proximal to *v* is merged to *v* by removing *u* from the graph by updating the two adjacency lists such that the incoming and outgoing array entries storing pointers to *u* are updated to store pointers to *v*. This can be achieved in linear time. We repeat this until all proximal nodes have been merged. Figure 3 illustrates the construction of the bi-labelled de Bruijn graph for a pair of Rmaps.

**Fig. 4:**
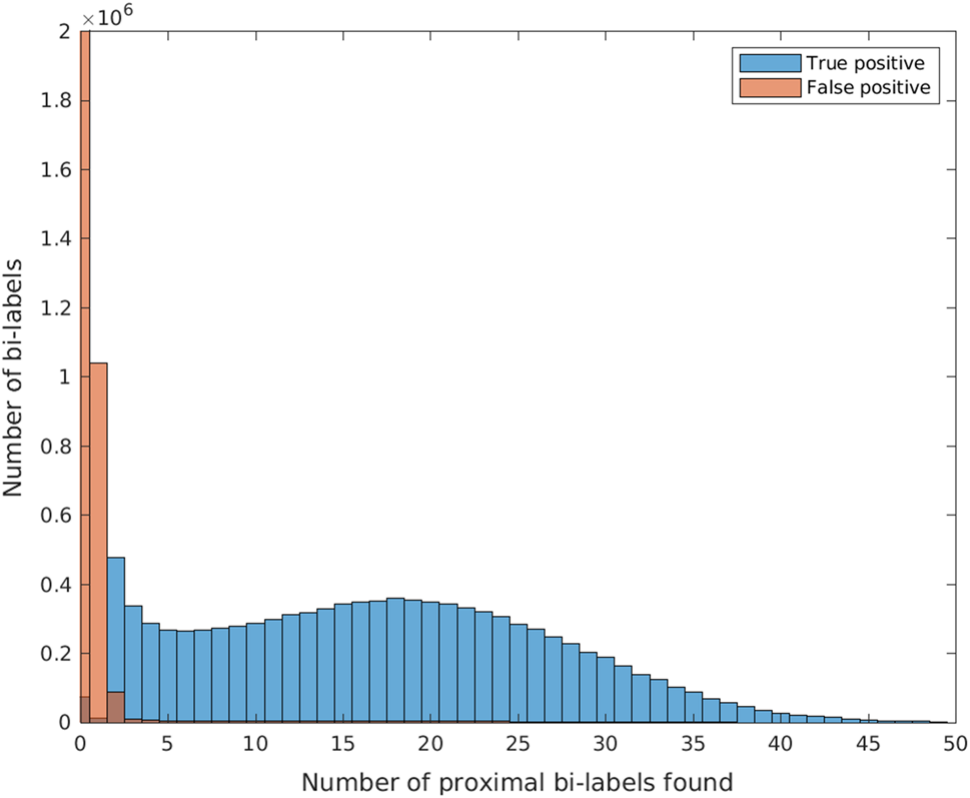
Histogram showing the precision of finding proximal bi-labels. For simulated human Rmap data, we found proximal bi-labels for all extracted bi-labels. We designate a proximal bi-label found to be a true positive if its true location in the genome is the same as the location of the bi-label to which it is proximal—and false positive otherwise. Next, we plotted a histogram showing the distribution of true positives and false positive proximal bi-labels for each bi-label. We show that high frequency bi-labels i.e. bi-labels for which we find more proximal bi-labels produce more precise proximal bi-labels. This justifies filtering low frequency bi-labels

### Graph cleaning and traversal

Before traversing the graph, we first pre-process the bi-labelled de Bruijn graph to remove *tips* and *bubbles*, which are common in de Bruijn graphs. Since they limit the size of unary paths (i.e. paths in the graph that contain nodes with only a single outgoing edge) and do not affect the accuracy of the assembly, it is common practice in short read assembly to resolve or remove these structures [33–36]. Tips are produced when errors cause an otherwise unary path to branch at a node and create a short unary path that ends in a terminal node. Bubbles are created when bi-labels from the same genomic location are not merged and included in the graph as separate edges. This generates short unary paths that have the same starting node and the same ending node and are close in length.

Similar to existing short read assemblers, we identify all tips and bubbles that have length of at most a specified threshold by performing depth first search starting at each node with out-degree greater than one. Hence, if there exists a tip starting at a given node as well as a path of length longer than the specified threshold, then the tip is removed by deleting all of its edges starting at the branching node. Furthermore, if there exists a bubble starting at a given node, we remove one of the edges adjacent to the branching node. We note we do not remove an entire path from the graph to resolve a bubble—rather, we only disconnect them at the branching node. Following the work of Simpson et al. [35], we fix the maximum length of the paths in a bubble to twice the size of the bi-label.

After cleaning, our traversal algorithm extracts unitigs (i.e. contigs corresponding to unary paths) from the graph by performing a simple depth first traversal starting from each node with zero incoming edges. We terminate the traversal of a given path if a cycle is reached or a node with out-degree greater than one is reached.

## Experiments

In this section, we compare the performance of ${\textsc {rmapper}}$, the assembler of Valouev et al. and Bionano Solve. We used the most recent version of Bionano Solve that is publicly available (version 3.5.1.). We performed all experiments on Intel E5-2698v3 processors with 192 GB of RAM running 64-bit Linux. Valouev and ${\textsc {rmapper}}$ were ran on error corrected data, which is analogous to assembly of sequence reads. Bionano Solve was not because the input is required to be specified in their proprietary format. In addition, for larger genomes, we also ran ${\textsc {rmapper}}$ by extracting bi-labels from both directions in an Rmap. We refer to this as ${\textsc {rmapper}}$2.0.

For all experiments we report the run time (CPU time), peak memory, maximum and mean contig size, genome fraction and number of mis-assembled contigs. We note that genome assembly evaluation tools such as QUAST [37] cannot be used on optical maps—hence, we design our own evaluation setup. To compute the genome fraction, we align all assembled contigs to the optical map reference genome using the alignment method of Valouev et al. [25]. The optical map reference genome is produced by *in silico* digesting the reference genome using the same restriction enzyme as used for producing the Rmaps. For all contigs that were successfully aligned, we designate their alignment locations on the reference genome as covered and report the percentage of the genome covered by at least one contig as the genome fraction. Any contig which is unable to be aligned by Valouev et al. is verified to be mis-assembled by aligning it to the reference genome using a second alignment software—Bionano’s RefAligner. The Valouev method aligns an assembled contig to a contiguous stretch of the reference optical map that optimizes its alignment score and does not tolerate mis-assembled regions, whereas RefAligner allows split alignments. Hence, if the alignment outputted from RefAligner is uncontiguous then it is counted as a mis-assembly.

rmapper takes as input four parameters, namely the size *k* of the *k*-mers, the minimum distance *D* between the two *k*-mers in the bi-label, and the error tolerance parameter setting $t_{f}$ and $t_{\ell }$. The *k*-mer size depends on the rate of added and missed cut-sites in the Rmap data. When the frequency of added and missed cut-sites is high, the *k*-mer size needs to be set low so that a good percentage of *k*-mers are error-free. We note that the average error-rate of optical-map data typically lies around 17% [30]. Considering that error-correction of the Rmaps is likely to bring the average error-rate below 10% [31], the *k*-mer size of 6 is the largest value such that the probability that an extracted *k*-mer will be error-free is at least $50\%$. Hence we use 6 as the default *k*-mer size in our experiments. The best combination of coverage, average length of contigs and run-time is achieved by fixing $t_{\ell } = {2000}$. We experimented with the following values of $D = \{{15000}, {20000}, {25000}, {30000}\}$ and the following values of $t_f = \{{500}, {1000}, {1500}\}$ and for each experiment, we choose the parameter setting that gives the best performance. A higher value of $t_f$ is needed when the Rmap data still has significant sizing errors after error correction. A lower value of *D* is needed when the average Rmap size is small so that we can extract an adequate number of bi-labels from each Rmap. We show the impact of varying the parameters on the *E. coli* genome in Section *Impact of parameters*.

### Datasets

We performed experiments on both simulated and real Bionano data. We simulated data from both *E. coli* K-12 substr. MG1655 genome and the human reference genome GRCh38 (NCBI accession number GCF_000001405.26) with OMSim [38]. We used enzyme BspQI — a standard, commonly used restriction enzyme for optical mapping — and used the default error rate of OMSim, which is a 15% rate of deleted cut sites, and 1 added cut site per 100kbp. The resulting *E. coli* dataset contains 23450 Rmaps with a mean of 42 fragments per Rmap. The human dataset contains 377894 Rmaps with a mean of 61 fragments per Rmap.

Lastly, we performed experiments using the Rmap dataset of the climbing perch (*Anabas testudineus*) genome generated for the Vertebrate Genomes Project, which consists of 3121480 Rmaps with mean of 28 fragments. A draft assembly of the genome is provided from the same source which was used to obtain the reference genome optical map.

### Impact of parameters

**Fig. 5:**
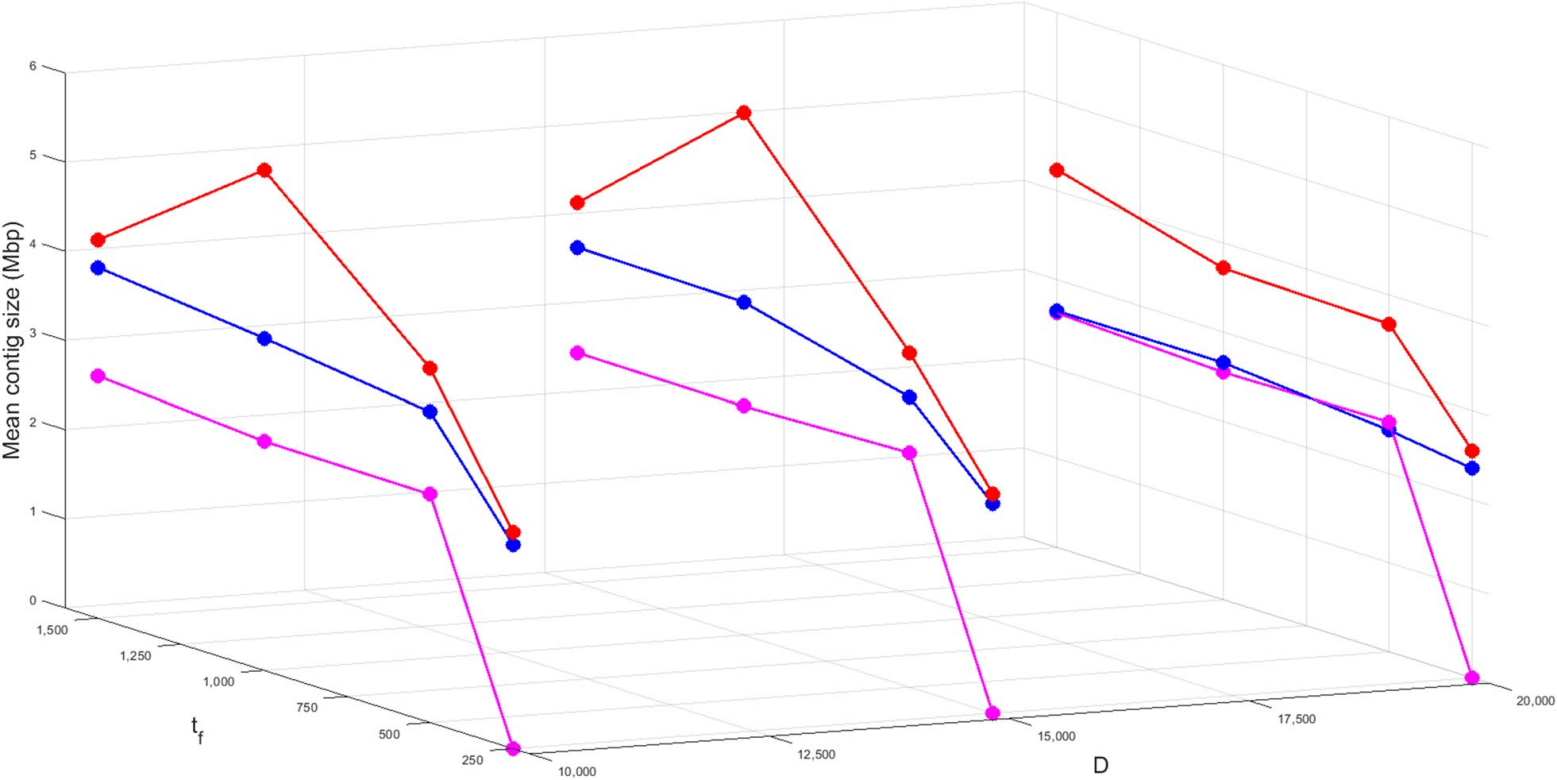
Impact of varying parameters *k*, *D*, and $t_f$ on the assembly of *E*. ***coli***. For all possible combination of these parameters, we calculated and reported the mean contig size. The blue lines depict a *k*-mer size 5, the red lines depict a *k*-mer size 6, and the magenta lines depict a *k*-mer size 7

We investigated the impact of parameters on assembly results of *E. coli* by varying the *k*-mer size, the parameter *D* (which denotes the length of the skip segment, the parameter $t_f$, and the parameter $t_l$. We considered the following set of values for these parameters: $k = \{5,6,7\}$, $D = \{{10000}, {15000}, {20000}\}$, $t_f = \{{250}, {500}, {1000}, {1500}\}$, and $t_l =\{{1500}, {2000}, {3000}\}$. We show the impact of varying *k*, *D* and $t_f$ in Fig. 5. The detailed statistics of this experiment are found in Additional file 1: Table S1. For this experiment, $t_l$ was fixed at 2,000. In Table 1, we show the impact of varing $t_f$ and $t_l$ together. For all experiments, contigs longer than 250 fragments are reported. The experiments show that for $t_f=250$ the assembly quality is poor. This is justified since the average sizing error exceeds 250. Similarly, for increasing values of *D*, we see a drop in the quality. This is because larger values of *D* create fewer number of bi-labels from an Rmap which reduces the effective coverage of the data. Among the three *k*-mer sizes used, best assembly quality is achieved with $k=6$. This is set as our default *k*-mer value for all experiments.

**Table 1:** Impact of varying the values of $t_f$ and $t_l$ on the assembly results for *E. coli* data

$t_f$	$t_l$	Run time(s)	Peak Memory(Mb)	No. of contigs	Max	Mean
250	1500	179	305	4	272 (2.420 Mbp)	267 (2.326 Mbp)
250	2000	200	305	3	271 (2.418 Mbp)	270 (2.351 Mbp)
250	3000	232	305	3	271 (2.419 Mbp)	267 (2.333 Mbp)
500	1500	403	459	10	336 (3.072 Mbp)	295 (2.625 Mbp)
500	2000	445	459	23	529 (4.701 Mbp)	371 (3.252 Mbp)
500	3000	509	459	49	529 (4.711 Mbp)	430 (3.793 Mbp)
1,000	1500	476	628	33	531 (4.745 Mbp)	427 (3.792 Mbp)
1,000	2000	533	629	29	529 (4.746 Mbp)	422 (4.746 Mbp)
1,000	3000	629	630	35	530 (4.742 Mbp)	412 (3.662 Mbp)
1,500	1500	537	705	11	424 (3.732 Mbp)	347 (3.028 Mbp)
1,500	2000	616	709	28	533 (4.778 Mbp)	424 (3.760 Mbp)
1,500	3000	748	711	22	535 (4.764 Mbp)	440 (3.887 Mbp)

In this Table, the value of *k* was fixed to 6, and the value of *D* was fixed to 15,000. The contig with maximum length (Max) is reported in the number of fragments and the total genomic length in mega base pairs (Mbp). Similarly, the mean contig length (Mean) is also reported in the number of fragments and the total genomic length in mega base pairs

### Performance on* E. coli*

For the *E. coli* Rmap dataset, error correction took 2.66 hours of CPU time. The assembly results are summarized in Table 2. For this experiment we extracted bi-labels with $k=6$ and $D={15000}$ and used error tolerance parameter setting $t_{f}={500}$ and $t_{\ell }={2000}$. rmapper took 342 seconds and peak memory of 274 Mb to assemble the data. The assembler produced two unitigs longer than 500 fragments, that are 529 and 522 fragments in length, both of which covered the reference from start to finish.

**Table 2:** Assembly results for* E. coli* Rmap data simulated by OMSim using enzyme BspQI

Assembler	Run time	Peak Memory	No. of contigs	Max	Mean	GF(%)	MA
Valouev	8.5 d	0.48	5	102 (1.0 Mbp)	56 (0.5 Mbp)	48	0
Solve	48.1 h	1.18	1	631 (4.9 Mbp)	631 (4.9 Mbp)	100	0
${\textsc {rmapper}}$	6 m	0.46	2	529 (4.6 Mbp)	526 (4.5 Mbp)	100	0

The dataset has 23,450 Rmaps of mean size of 42 fragments and coverage of 900x. The peak memory is given in gigabytes (GB). The run time is reported in second (s) minutes (m), hours (h) and days (d). rmapperwas run with $k=6$, $D={15000}$ and error tolerance parameter setting $t_{f}={500}$ and $t_{\ell }={2000}$. The contig with maximum length (Max) is reported in the number of fragments and the total genomic length in mega base pairs (Mbp). Similarly, the mean contig length (Mean) is also reported in the number of fragments and the total genomic length in mega base pairs. The genome fraction (GF) is the percentage of the genome that is covered by at least one contig. Lastly, the number of mis-assembled contigs (MA) is given

The Valouev assembler [24] took 204.8 hours to compute pairwise alignments between all pairs of Rmaps and an additional 30 minutes to assemble them into contigs. It produced 5 contigs with the longest contig of length 102 fragments (corresponding to a 1Mbp genomic span). We aligned the assembled contigs back to the reference and found the total genome coverage to be 48%. Bionano solve produced a high quality assembly, i.e., one contig that spanned 100% of the genome. The assembly took 48.14 hours of CPU time (59.75 minutes of wall time using 60 CPUs in parallel) and peak memory of 1.18 GB. The Valouev aligner reported alignments for all contigs, hence we report zero mis-assembled contigs for all three methods.

In summary, the quality of Bionano Solve and ${\textsc {rmapper}}$ were comparable, yet ${\textsc {rmapper}}$ was 480 times faster (6 minutes versus 2889 minutes) and used less than 500 Mb of memory.

### Performance on human

For the human Rmap dataset, error correction took 1339.31 seconds of wall time running cOMet in parallel on 2000 CPUs (corresponding to 524 hours of CPU time). The assembly results are shown in Table 3. For this experiment we extracted bi-labels with $k=6$ and $D={25000}$ and used error tolerance parameter setting $t_{f}={1500}$ and $t_{\ell }={2000}$. rmapper took 12.1 hours and peak memory of 7.9 GB to assemble the data whereas rmapper 2.0 took 22.2 hours and 18.8 GB of peak memory. rmapper produced 3134 contigs whereas ${\textsc {rmapper}}$ 2.0 produced 2867 contigs. The maximum size unitig produced by ${\textsc {rmapper}}$ and ${\textsc {rmapper}}$2.0 was 1380 and 1752 fragments in length, respectively. Lastly, rmapper achieved a net coverage of 95.8% while rmapper2.0 was able to cover 96.7% of the genome—both with zero mis-assembled contigs.

**Table 3:** Assembly results for human Rmap data simulated by OMSim using enzyme BspQI

Assembler	Run time	Peak Memory	No. of contigs	Max	Mean	GF(%)	MA
Valouev	$> 360$ d	n/a	n/a	n/a	n/a	n/a	n/a
Solve	122.4 d	94.8	169	14,133 (124.6 Mbp)	2,036 (16.4 Mbp)	93.8	4
rmapper	12.1 h	7.9	3865	1,380 (14.4 Mbp)	144 (1.4 Mbp)	95.8	0
${\textsc {rmapper}}$ 2.0	22.2 h	18.8	3524	1,752 (18.5 Mbp)	203 (2.0 Mbp)	96.7	0

The dataset has 377894 Rmaps of mean size of 61 fragments and coverage 80x. See Table 2 for a description of the assembly statistics and notation. As described in the text, rmapper2.0 extracts bi-labels from Rmaps in both forward and reverse directions

The Valouev assembler did not produce any output after 360 CPU days so n/a is reported in Table 3. Bionano Solve produced comparably fewer but longer contigs to ${\textsc {rmapper}}$ but had 4 mis-assembled contigs. In addition, it took approximately 2937 CPU hours (55 hours of wall time using 60 CPUs in parallel) and peak memory of 94.8 GB. It is also worth noting that Bionano Solve performs an elaborate scaffolding and stitching of contigs, which explains the relatively few number of contigs but higher mis-assembly rate. The scaffolding and stitching cannot be decoupled from the assembly since Bionano only distributed a single executable that runs both. The source code is not publicly available.

In summary, the Valouev assembler did not scale to the human genome, ${\textsc {rmapper}}$2.0 produced slightly longer contigs than ${\textsc {rmapper}}$, Bionano Solve produced the longest contigs but covered 93.8% of the genome and had 4 mis-assembled contigs. In addition, ${\textsc {rmapper}}$2.0 has the highest genome fraction, which is 96.7%. Lastly, ${\textsc {rmapper}}$ and ${\textsc {rmapper}}$2.0 was 242 and 132 times faster than Solve, respectively, and used 5 times less memory.

### Performance on climbing perch

Error correction of the the climbing perch (*Anabas Testudineus*) Rmap dataset took 1.84 hours of wall time running cOMet in parallel on 3000 CPUs (corresponding to 2042 hours of CPU time). The assembly results are shown in Table 4. For this experiment we extracted bi-labels with $k=6$ and $D={15000}$ and used error tolerance parameter setting $t_{f}={1500}$ and $t_{\ell }={2000}$. rmapper took 7.5 hours and peak memory of 9.7 GB to assemble the data whereas rmapper 2.0 took 14.9 hours and 18.77 GB of peak memory. rmapper produced 4573 contigs whereas ${\textsc {rmapper}}$ 2.0 produced 4972 contigs. The maximum size unitig produced by ${\textsc {rmapper}}$ and ${\textsc {rmapper}}$ 2.0 was 217 and 294 fragments in length, respectively. Lastly, rmapper achieved a genome fraction of 92.07%, while rmapper 2.0 was able to cover 95.05% of the genome. Both rmapper and rmapper2.0 produced zero mis-assemblies.

**Table 4:** Assembly results for the Rmap dataset of the climbing perch genome

Assembler	Run time	Peak Memory	No. of contigs	Max	Mean	GF(%)	MA
Solve	156 d	16 Gb	907	1032 (8.4 Mbp)	104 (7.9 Mbp)	97.6	5
${\textsc {rmapper}}$	7.5 h	9.7	4573	217 (1.6 Mbp)	32 (0.28 Mbp)	92.07	0
${\textsc {rmapper}}$2.0	14.9 h	18.8	4972	294 (2.4 Mbp)	42 (0.4 Mbp)	95.05	0

The data was generated for the Vertebrate Genomes Project and it consists of 3121480 Rmaps with mean size of 28 fragments. The restriction enzyme used in the experiment is BspQI. See Table 2 for a description of the assembly statistics and notation. As described in the text, rmapper2.0 extracts bi-labels from Rmaps in both forward and reverse directions. Bionano Solve halted with a fatal error message in its final scaffolding step. We used the latest assembly result produced by the Solve in order to compare their assembly quality

The Valouev assembler did not halt on this dataset after 360 CPU days so we do not report any results. Solve halted with a fatal error message in its final scaffolding step after 156 CPU days (93 hours of wall time using 60 CPUs in parallel) and using a peak memory of 16 GB. We used the latest assembly result produced by the software in order to compare their assembly quality. Similar to the human assembly, Bionano Solve produced comparably fewer and longer contigs to ${\textsc {rmapper}}$ and had a genome coverage of 97.8%—but had 5 mis-assembled contigs. In summary, the Valouev assembler did not scale to the human genome, ${\textsc {rmapper}}$2.0 produced slightly longer contigs than ${\textsc {rmapper}}$, Bionano Solve produced the longest contigs and covered 97.6% of the draft genome but had 5 mis-assembled contigs. ${\textsc {rmapper}}$2.0 has comparable genome coverage to Solve, which is 95.05% — while running 251 times faster.

## Discussion and future work

We implement our approach and show its performance on multiple simulated and real datasets. Our experimental results show the only non-proprietary method (i.e. by Valouev et al. [24]) is unable to scale to the human and fish genomes, and that our method is at least 130 times faster than Bionano Solve and its memory usage is less than 20% of the memory usage of Bionano Solve. We point out that there is a trade-off between the length of the contigs, the genome fraction, and number of mis-assemblies. Analogous to assembly of short reads, ideally an assembler should return a small number of contigs or scaffolds which cover the entire genome and have no mis-assembled regions. In the case of the human and fish data, Solve was able to produce fewer and longer scaffolds than ${\textsc {rmapper}}$ but produced more mis-assemblies than ${\textsc {rmapper}}$. Conversely, for the human data, ${\textsc {rmapper}}$ produced contigs that covered a larger fraction of the genome with no mis-assembled regions. This highlights one trade-off in Rmap assembly. Hence, there is an opportunity to improve Rmap assemblers so that this gap between Solve and ${\textsc {rmapper}}$ is closed. Another important note about the comparison between the assemblers is that ${\textsc {rmapper}}$ has a very simple traversal algorithm and does not use any sort of scaffolding. This is due to the fact that the main contribution of this work is formulating and solving the problem of assembly of Rmaps. Bionano Solve has a scaffolding algorithm that cannot be decoupled from the assembly step since only an executable is available. Thus, the results really compare ${\textsc {rmapper}}$’s unitigs with Solve’s scaffolds, and ${\textsc {rmapper}}$ is still comparable.

This work presents the first non-proprietary Rmap assembler developed in the past decade, and thus, opens the door for improving Rmap assembly. Thus, there are many related problems and possible improvements that warrant future research. First, the main contribution of our work was adapting the de Bruijn graph to Rmap data. For completeness, we perform depth first search to traverse the bi-labelled de Bruijn graph and extract contigs. Our traversal does not attempt to reconcile complicated regions in the graph, however, we believe that there is a great opportunity to improve the length of the assembled optical maps by devising an algorithm to extend the traversal. Next, we hypothesize that by adapting methods designed for scaffolding and stitching optical mapping data [39, 40], the length of the assembled optical maps can be improved. Lastly, we note that there does not exist a method to evaluate optical map assembles like there does for genome assemblies—QUAST [37] being the well-known genome assembly evaluation method. Furthermore, although some of the metrics of genome assembly evaluation tools (e.g., mean contig length and length of the longest contig) trivially extend to optical map assemblies, metrics that require sequence alignment to a reference genome (e.g., number of mis-assemblies) do not extend and need redevelopment.

## Conclusion

Assembly of Rmap data is a fundamental problem in optical mapping that still remains in a nascent stage—as prior to this work, there was only a single other non-proprietary assembler. In this paper, we formulate and describe the first de Bruijn graph approach for Rmap assembly by redefining the de Brujn graph to adapt it to Rmap data. We accomplish this by extending the definition of a bi-label introduced in the context of the paired-end de Bruijn graph by Medvedev et al. [28]. We refer to our modified de Bruijn graph as the bi-labelled de Bruijn graph and demonstrate how to efficiently build and store it using a two-tiered orthogonal range search data-structure.

We implement this approach, leading to a novel Rmap assembler that we call ${\textsc {rmapper}}$. We compare the performance of our method with the assembler of Valouev et al., and Bionano Solve on three genomes of varying size: *E. coli*, human, climbing perch (a fish species from the Vertebrate Genomes Project). Our comparison demonstrates that ${\textsc {rmapper}}$ was more than 130 times faster and used less than five times less memory than Solve, and was more than 2,000 times faster than Valouev et al.. Consequently, ${\textsc {rmapper}}$ successfully assembled the 3.1 million Rmaps of the climbing perch genome into contigs that covered over 95% of the draft genome with zero mis-assemblies.

## Supplementary Information

**Additional file 1: Table S1.** Impact of varying the values of *k*, *D* and *t*_f_ on the assembly results for *E. coli* data.

## Data Availability

Our software, rmapper is written in C++ and is publicly available under GNU General Public License at https://github.com/kingufl/Rmapper. Additional data can be accessed from the Github repository.
