# Supplementary material for: Fast and efficient Rmap assembly using the Bi-labelled de Bruijn graph
Source: Algorithms Mol Biol. 2021 May 25;16:6. doi: 10.1186/s13015-021-00182-9 (PMC8147420; doi:10.1186/s13015-021-00182-9)
Supplement: Supplementary file 1 — Additional file 1: Table S1. Impact of varying the values of k, D and tf on the assembly results for E. coli data. [file 13015_2021_182_MOESM1_ESM.pdf]

Supplement - Fast and Efficient Rmap Assembly using the Bi-labelled de Bruijn Graph

Kingshuk Mukherjee, Massimiliano Rossi, Leena Salmela, Christina Boucher

Table 1: Impact of varying the values of k, D and t_f on the assembly results for *E. coli* data. In this Table, the value of k was fixed to 6, and the value of D was fixed to 15,000. The contig with maximum length (Max) is reported in the number of fragments and the total genomic length in mega base pairs (Mbp). Similarly, the mean contig length (Mean) is also reported in the number of fragments and the total genomic length in mega base pairs.

**kmer size**	**D size**	**t_f**	**Run time (s)**	**Peak Memory (Mb)**	**Number of contigs**	**Max**	**Max length (Mbp)**	**Mean**	**Mean length (Mbp)**
5	10,000	250	482	475	19	340	3.042	277	2.428
5	10,000	500	689	733	42	533	4.701	452	3.973
5	10,000	1000	924	986	42	983	8.587	642	5.613
5	10,000	1500	1055	1102	37	543	4.786	482	4.243
5	15,000	250	429	447	18	338	3.036	280	2.466
5	15,000	500	638	688	43	533	4.701	428	3.752
5	15,000	1000	863	924	41	975	8.565	666	5.855
5	15,000	1500	986	1031	34	537	4.763	486	4.265
5	20,000	250	399	420	9	332	2.901	294	2.55
5	20,000	500	586	639	41	530	4.676	419	3.674
5	20,000	1000	788	857	33	537	4.754	423	3.716
5	20,000	1500	915	952	36	544	4.764	482	4.236
6	10,000	250	294	320	5	272	2.425	264	2.287
6	10,000	500	426	491	21	533	4.704	402	3.485
6	10,000	1000	586	673	34	529	4.745	420	3.726
6	10,000	1500	663	755	27	533	4.754	444	3.934
6	15,000	250	273	305	3	271	2.418	270	2.351
6	15,000	500	393	459	23	529	4.7	371	3.252
6	15,000	1000	534	629	29	529	4.746	422	3.734
6	15,000	1500	614	709	28	533	4.778	424	3.76
6	20,000	250	254	287	3	272	2.448	268	2.35
6	20,000	500	367	427	7	335	2.929	290	2.486
6	20,000	1000	490	581	5	335	2.929	305	2.656
6	20,000	1500	562	654	3	334	2.929	306	2.65
7	10,000	250	183	228	0	0	0	0	0
7	10,000	500	261	330	6	335	3.001	292	2.561
7	10,000	1000	348	445	10	336	3.031	293	2.571
7	10,000	1500	396	499	5	338	3.044	307	2.718
7	15,000	250	171	217	0	0	0	0	0
7	15,000	500	240	310	6	335	2.955	303	2.63
7	15,000	1000	322	416	7	335	2.958	298	2.566
7	15,000	1500	368	466	6	335	3.022	298	2.578
7	20,000	250	157	207	0	0	0	0	0
7	20,000	500	219	288	7	333	2.985	295	2.579
7	20,000	1000	292	384	6	335	2.985	294	2.548
7	20,000	1500	332	426	5	337	3.002	301	2.627
